# Interplay of negative electronic compressibility and capacitance enhancement in lightly-doped metal oxide Bi_0.95_La_0.05_FeO_3_ by quantum capacitance model

**DOI:** 10.1038/s41598-020-61859-6

**Published:** 2020-03-20

**Authors:** S. Nathabumroong, T. Eknapakul, P. Jaiban, B. Yotburut, S. Siriroj, T. Saisopa, S.-K. Mo, R. Supruangnet, H. Nakajima, R. Yimnirun, S. Maensiri, W. Meevasana

**Affiliations:** 1 School of Physics and Center of Excellence on Advanced Functional Materials, Suranaree University of Technology, Nakhon Ratchasima, 30000 Thailand; 2 Faculty of science, Energy and Environment, King Mongkut’s University of Technology North Bangkok, Rayong Campus, Rayong, 21120 Thailand; 3 Advanced Light Source, Lawrence Berkeley National Laboratory, Berkeley, CA 94720 USA; 4 Synchrotron Light Research Institute, Nakhon Ratchasima, 30000 Thailand; 5 School of Energy Science and Engineering, Vidyasirimedhi Institute of Science and Technology, Rayong, 21210 Thailand; 6 Thailand Center of Excellence in Physics (ThEP), MHSRI, Bangkok, 10400 Thailand

**Keywords:** Ceramics, Surfaces, interfaces and thin films, Electronic properties and materials, Ferroelectrics and multiferroics

## Abstract

Light-sensitive capacitance variation of Bi_0.95_La_0.05_FeO_3_ (BLFO) ceramics has been studied under violet to UV irradiation. The reversible capacitance enhancement up to 21% under 405 nm violet laser irradiation has been observed, suggesting a possible degree of freedom to dynamically control this in high dielectric materials for light-sensitive capacitance applications. By using ultraviolet photoemission spectroscopy (UPS), we show here that exposure of BLFO surfaces to UV light induces a counterintuitive shift of the O_2*p*_ valence state to lower binding energy of up to 243 meV which is a direct signature of negative electronic compressibility (NEC). A decrease of BLFO electrical resistance agrees strongly with the UPS data suggesting the creation of a thin conductive layer on its insulating bulk under light irradiation. By exploiting the quantum capacitance model, we find that the negative quantum capacitance due to this NEC effect plays an important role in this capacitance enhancement

## Introduction

Bismuth Ferrite (BiFeO_3_) is a multiferroic oxide material which has been extensively studied due to its ability to simultaneously exhibit both magnetic and strong ferroelectric properties at room temperature^1,2^. As such, BiFeO_3_ has recently drawn much interest in potential applications spanning spintronics, magnetoelectric sensors and photovoltaic devices^3–5^. Several studies also attempt to enhance the dielectric constant of BiFeO_3_ which could effectively improve its ferroelecticity^6^. In fact, a pure BiFeO_3_ is difficult to synthesize whose dielectric constant was reported only 50–100 at 10 kHz^7,8^. Slight modifications to BiFeO_3_ ceramics have been reported featuring both giant dielectric constant (>10^4^ at room temperature) and sufficiently low dissipation factor^9,10^ to be suitable for magnetodielectric applications^11^. Recently, efforts to improve the dielectric behavior of BiFeO_3_ have been reported, such as varying preparation methods^12^ and dopants^13^. Such extrinsic dielectric constant enhancement can be controlled by lattice distortions, particle sizes, domains, or impurities^12,14^.

In addition to extrinsic dielectric constant enhancement, the capacitance of oxide materials can intrinsically be improved by tuning carrier densities (i. e. n-type doping or applied gate electric field). Such dielectric tunabilities have potential applications as various microwave devices, such as phase shifters and varactors^15^. Recently, capacitance enhancement at the LaAlO_3_/SrTiO_3_ interface in excess of 40% was found to originate from negative electron compressibility (NEC) at low electron density (n)^16^, enabled as the accumulation of all mobile electrons in the interfacial region which make quantum conduction and therefore quantum capacitance is the dominant model^16–18^. The negative thermodynamic density of state ($\frac{dn}{d\mu } < 0$), where *μ* is chemical potential, has been observed in several 2D materials and interfaces^16,19^, carbon nanotubes^20^, and bulk materials^21^. Hence, researching and modifying materials having this NEC behavior could effectively enhance their capacitances, presenting alternatives to the use of high dielectric materials for nanoscale devices.

Alternatively, carrier densities on metal oxide surfaces can be controlled by the creation of oxygen vacancy states induced by light irradiation^22–25^. Recently, the capacitance enhancement induced by surface charge accumulation has been reported on CaCu_3_Ti_4_O_12_^26^. This is similar to the case of applying electric field (i. e. introduction of the quantum conductive interfaces) which might indicate the analogous microscopic origin. In this work, we observe a striking capacitance enhancement in lightly-doped metal oxide Bi_0.95_La_0.05_FeO_3_ (BLFO) under 405 nm violet laser irradiation. By using ultraviolet photoemission spectroscopy (UPS) and transport measurements, a signature of NEC has been revealed. By light irradiation, the experimentally-observed changes indicate that the quantum capacitance model plays a major role, which therefore supports claims for a strong interplay between NEC and capacitance enhancement. These findings are critical in understanding the fundamental nature of such system as well as establishing a new synthetic route to light-sensitive capacitive devices.

## Methods

### Sample preparation and characterisation

Our BLFO polycrystals were prepared by a simple co-precipitation method^9^. The dried precursors were calcined in air at 600 °C for 3 h. Sample powders were pressed into pellets with 1 cm diameter and sintered at 800 °C in air for 3 h. A small decrease of grain size (compared to the pure BiFeO_3_) and structural distortion were revealed in BLFO samples by X-ray diffraction (XRD) and scanning electron microscopy (SEM). The remarkable enhancement of BLFO capacitance might solely be affected by the reduction of electrical conductivity and leakage current^27^. We chose BLFO because of its high initial capacitance, for example, its dielectric constant was more than three times that of pure BiFeO_3_ ceramics prepared by the same method^9,14,28^. Details of sample preparation and characterisation are shown in Supplementary Information.

### Capacitance measurement

**Figure 1:**
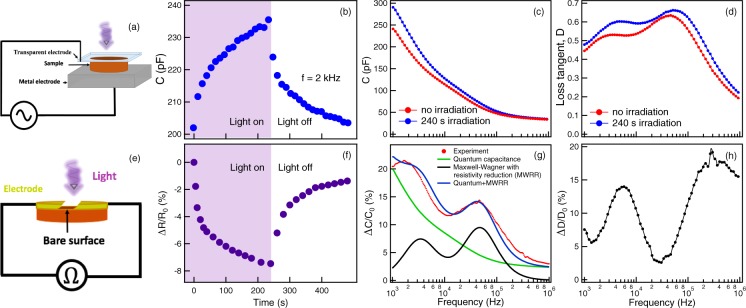
(**a**) Setup of capacitance measurement allowing light irradiation on the top electrode. (**b**) BLFO capacitance measured as a function of violet laser irradiation at f = 2 kHz with 480 s time scale. Frequency dependence of (**c**) capacitance and (**d**) loss tangent with and without violet laser irradiation. (**e**) Diagram of resistance measurement. (**f**) BLFO resistance measured under violet laser irradiation. (**g**) The change of capacitance after 240 s irradiation (red-dotted curve). Green and black curves represent the calculated capacitance enhancement using quantum capacitance and Maxwell-Wagner with resistivity reduction (MWRR) model, respectively. The summation of these model is represented by blue curve which is in agreement with the observed capacitance enhancement by light irradiation. (**h**) The change of loss tangent after 240 s irradiation.

Instead of a metal top electrode, our capacitor was fabricated with transparent conductive indium tin oxide (ITO) allowing light irradiation on this side. The BLFO sample was mechanically compressed against ITO and metal electrode as shown in Fig. 1(a). The capacitance measurements were performed using a standard impedance analyzer (Agilent: model 4294A) with alternating voltage output (V_*ac*_ = 0.5 V) and frequency ranging from 1 kHz to 1 MHz. A 405 nm laser corresponding to 3.06 eV photon energy (with fixed intensity of 1.8 W.cm^−2^ and 3 × 3 mm^2^ beamsize) was used throughout the experiment. This photon energy is smaller than the ITO optical band gap and work function of most materials (≈4eV), hence, competing effects occurring upon light irradiation such as light absorption and photoelectron ejection would not be expected^29^.

### Ultraviolet photoemission spectroscopy

To understand the microscopic mechanism that drives this light-sensitive behavior in BLFO, the electronic structure of the BLFO sample was measured by ultraviolet photoemission spectroscopy (UPS) using Scienta R4000 electron analyzer located at BL 10.0.1 of the Advanced Light Source (USA) and BL 3.2a of the Synchrotron Light Research Institute (SLRI), Thailand. The measurements were performed at room temperature with base pressure better than 5 × 10^−8^ mbar. Photon energy was set to be 60 eV with 0.3 × 0.1 mm^2^ beam size.

## Results and discussion

The BLFO capacitance measured at f = 2 kHz as a function of light irradiation is shown in Fig. 1(b). The initial capacitance measured in this setup is found to be around 200 pF. After irradiation for 240 s, the capacitance increased gradually and then became saturated at 236 pF. After turning off the irradiation, its capacitance decayed slowly and then reached the value close to the initial value. The frequency dependent measurements of capacitance and loss tangent before and after irradiation are shown in Fig. 1(c,d). The dielectric behaviour can be described by the Debye type equation including electrode and grain-boundary effects^30^.

Resistance measurement under light irradiation was performed on BLFO surfaces by preparing 1 mm-wide surface in between the gold electrodes (Fig. 1(e)). After turning on the laser for 240 s, BLFO resistance decreased by 8%. Similar to the capacitance measurement, its resistance recovers close to the initial value after a 240 s absence of irradiation (Fig. 1(f)). The features of the changes in capacitance and resistance suggest the existence of at least two effects occurring during the on-off process, including photogeneration of charge carriers (photoconductivity) and creation of oxygen vacancy. When light is on, photoconductivity is known to contribute to the changes in capacitance and resistance instantly and dynamically. It was found that both capacitance and resistance change quickly and immediately when turning off the light (at time ≈240 s of Fig. 1(b,f)) which could be described by the photogeneration of charge carriers. However, since the capacitance measured here does not instantly recover back to its original value when the irradiation is off, such changes are attributed to the creation of a thin conductive layer upon the insulating bulk referred to as a quantum-confined electron gas which is related to the creation of oxygen vacancies induced by light irradiation^26,31,32^.

The increases of capacitance and loss tangent are shown in Fig. 1(g,h). We found that the capacitance enhancement can be measured as high as 21% at frequency around 2 kHz after 240s of irradiation. The capacitance enhancement lineshape is nicely-fitted with incorporating of a reduction of surface resistivity (due to photogeneration of charge carrier) described by Maxwell-Wagner model^33^ (see Supplementary Information) and NEC effect described below.

**Figure 2:**
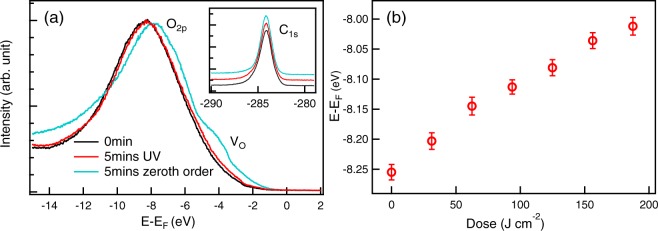
(**a**) Valence band spectra of BLFO ceramic measured at different conditions. The O_2*p*_ state shifts to lower binding energy while the C_1*s*_ (inset) is located at the same energy. (**b**) Summary of the O_2*p*_ position as a function of light dose.

The UPS spectrum of the fresh sample was measured immediately (i. e. 0 min of irradiation, the black spectrum in Fig. 2(a)). It was found that the O_2*p*_ state located at a binding energy around 6-8 eV was consistent with other metal oxides^25,26,34^ indicating a good electrical contact between our sample and a sample holder. A counterintuitive shift of the O_2*p*_ state to lower binding energy was initially observed by the first doping condition (5 min of UV light exposure) as illustrated by the red spectrum in Fig. 2(a). The UPS spectrum after zeroth order light irradiation, i. e., light with all frequencies, clearly indicates a significant shift of around 1 eV to lower binding energy (the blue spectrum in Fig. 2(a)). Moreover, an intergap peak emerged at around 4 eV binding energy which can be assigned to the oxygen vacancy state (V_*O*_)^23,26^. In contrast, the C_1*s*_ state was also measured immediately after each UV irradiation (h*ν* = 500 eV) which clearly indicates no binding energy shift of C_1*s*_ state (inset of Fig. 2(a)) confirming the distinctive character of BLFO sample. By using standard Gaussian fitting^35,36^, a continuous shift of O_2*p*_ state as a function of UV dosing was found to reach the value as high as 243 meV at a maximum UV dosing of 180 J.cm^−2^ as summarized in Fig. 2(b) (see Supplementary Information).

Capacitance enhancement induced by light irradiation has been studied in several metal oxides^26,37^. To explain this, possible scenarios such as filling of material’s mid-gap state^38^, enhancement of total charge carrier density^39^, and the creation of a two-dimensional electron gas at the surface^26,40^ as a result of photo illumination have been proposed. Regarding these corroborated with our observed UPS spectra, we introduce the quantum capacitance (C_*q*_) model to explain the capacitance enhancement in BLFO. This quantum capacitance model is a consequence of the Pauli principle which requires extra energy for filling a quantum well with electrons accumulated near the surface^18^.

**Figure 3:**
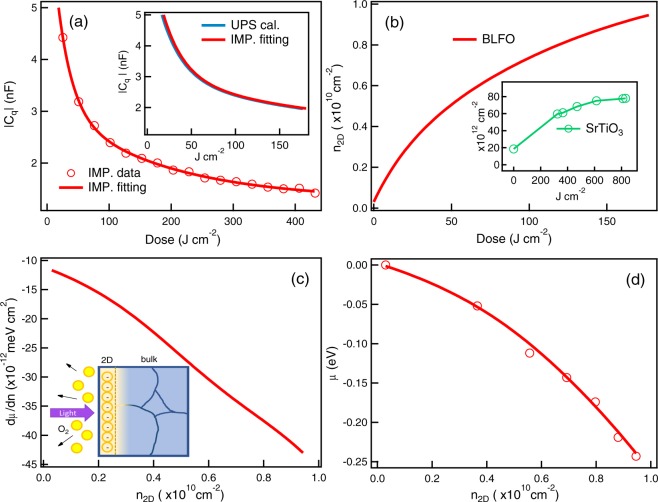
(**a**) C_*q*_ as a function of light dose. The calculated C_*q*_ between capacitance and UPS measurements is shown in the inset. (**b**) The calculated surface carrier density (n_2*D*_) as a function of light dose. For comparison of the line shape of *n*_2*D*_, we have added the n_2*D*_ measurement of SrTiO_3_ (inset) from ref. ^24^. (**c,d**) The increase of $\frac{d\mu }{dn}$ and *μ* as a function of n_2*D*_. All the circle symbols are from measurement and the lines are fit.

In our case, C_*q*_ formed on the irradiated-surfaces is manifested as capacitors in series with a geometric capacitance (*C*_*geo*_) between two electrical plates^41^. Total capacitance (C_*tot*_) can then be calculated by $\frac{1}{{C}_{tot}}=\frac{1}{{C}_{geo}}+\frac{1}{{C}_{q}}$, where C_*geo*_ = $\varepsilon \frac{A}{d}$ is solely dependent on the geometry^16,42^. *C*_*q*_ can be derived by the electron-electron interaction between layers (i. e. *C*_*q*_ = *C*_*kin*_ + *C*_*ex*-*corr*_) which represents capacitances due to kinetic and exchange-correlation energies respectively. Notably, C_*q*_ can be expressed by a term of thermodynamic density of states (${C}_{q}=A{e}^{2}\frac{dn}{d\mu }$)^16^ which strongly indicates that C_*q*_ can either be positive or negative depending on the sign of $\frac{dn}{d\mu }$^16,43^. From above equation, C_*tot*_ can be increased only in the case of negative C_*q*_ which means $\frac{dn}{d\mu } < 0$, whereby increasing the electron density leads to a decrease of chemical potential. In view of increasing C_*tot*_ as a function of light irradiation (Fig. 1b), the absolute value of measured C_*q*_ has been plotted by circle symbols in Fig. 3(a). Note that the similar values of C_*q*_ estimated from UPS spectra and impedance measurement are shown in the inset of Fig. 3(a).

According to the available information of both NEC and quantum capacitance, the creation of two-dimensional electron density (n_2*D*_ corresponding to n) upon UV light exposure can be estimated by ${n}_{2D}\approx \int \frac{{C}_{q}\frac{dV}{dD}}{Ae}.dD$^16^, where $V=\frac{\mu }{e}$ is the shift of O_2*p*_ state and D is light dose (see Supplementary Information). As shown in Fig. 3(b), the calculated n_2*D*_ increases as a function of light dosing reaching the value of 0.95 × 10^10^*cm*^−2^ at 180 J.cm^−2^. This exhibits similar trend with three orders of magnitude smaller than previous report of a light-irradiated surface of bulk SrTiO_3_ (reproducing data is shown in the inset of Fig. 3(b) with permission from ref. ^24^).

Regarding the expression of C_*q*_, $\frac{dn}{d\mu }$(or $\frac{d\mu }{dn}$) is found to be a crucial parameter which offers a quantitative capacitance calculation. As shown in Fig. 3(c), the calculated $\frac{d\mu }{dn}$ increases in negative values up to 43 × 10^−12^ meV cm^2^ at a maximum n_2*D*_ of 0.95 × 10^10^ cm^−2^. This value is about 2 times smaller than the previously observed 40% capacitance enhancement in LaAlO_3_/SrTiO_3_ interfaces^16^. A plot of negative chemical potential shift versus n_2*D*_ is shown in Fig. 3(d). Note that the circle symbols are the measured data taken from Fig. 2(b). This negative value can be described by the random phase approximation (RPA) for exchange and correlation interactions where the negative compressibility (up to 600 meV) can happen in two-dimensional electron system^19,44^. Overall, we note that our observed NEC and the capacitance enhancement is generally described by the creation of two-dimensional electron layer on BLFO ceramic^16,45^ induced by light irradiation.

## Conclusion

We have observed an increase of BLFO capacitance by up to 21% by visible light irradiation. Our experimental UPS data indicate creation of oxygen vacancies and the counterintuitive shift to lower binding energy on BLFO surfaces. The calculated negative values of *C*_*q*_ and the increase of n_2*D*_ under light irradiation suggest an emerging of negative quantum capacitance in our system which leads to the enhancement of overall capacitance. We have also demonstrated a presence of negative $\frac{d\mu }{dn}$ induced by light irradiation which provides an important role in driving such mechanism in two-dimensional electron system. Finally, our findings could create the new pathway in the study of the three-coupled degrees of freedom, i.e. light, charge and spin. Moreover, the capacitance enhancement in BLFO (multiferroics) could also help in the magnetoelectric/magnetocapacitance research and application.

## Supplementary information

Supplementary Information.

## References

[1] Catalan G, Scott JF (2009). Physics and applications of bismuth ferrite. Adv. Mater..

[2] Lebeugle D (2007). Room-temperature coexistence of large electric polarization and magnetic order in BiFeO_3_ single crystals. Phys. Rev. B.

[3] Baek SH (2010). Ferroelastic switching for nanoscale non-volatile magnetoelectric devices. Nat. Mater..

[4] Yang SY (2010). Above-bandgap voltages from ferroelectric photovoltaic devices. Nat. Nanotechnol.

[5] Yang SY (2009). Photovoltaic effects in BiFeO_3_. Appl. Phys. Lett..

[6] Paik H, Hwang H, No K (2007). Room temperature multiferroic properties of single-phase (Bi_0.9_ La_0.1_)FeO_3_ -Ba(Fe_0_. 5 Nb_0.5_)O_3_ solid solution ceramics. Appl. Phys. Lett..

[7] Kumar MM, Palkar VR, Srinivas K, Suryanarayana SV (2000). Ferroelectricity in a pure BiFeO_3_ ceramic. Appl. Phys. Lett..

[8] Lim S-H (2008). Enhanced dielectric properties in single crystal-like BiFeO_3_ thin films grown by flux-mediated epitaxy. Appl. Phys. Lett..

[9] Yotburut B, Yamwong T, Thongbai P, Maensiri S (2014). Synthesis and characterization of coprecipitation-prepared La-doped BiFeO_3_ nanopowders and their bulk dielectric properties. Jpn. J. Appl. Phys..

[10] Lin P, Cui S, Zeng X, Huang H, Ke S (2014). Giant dielectric response and enhanced thermal stability of multiferroic BiFeO_3_. J. Alloy. Comp..

[11] Markiewicz E, Hilczer B, Blaszyk M, Pietraszko A, Talik E (2011). Dielectric properties of BiFeO_3_ ceramics obtained from mechanochemically synthesized nanopowders. J. Electroceramics.

[12] Saxena P, Kumar A, Sharma P, Varshney D (2016). Improved dielectric and ferroelectric properties of dual-site substituted rhombohedral structured BiFeO_3_ multiferroics. J. Alloy. Comp..

[13] Li Y, Cao W-Q, Yuan J, Wang D-W, Cao M-S (2015). Nd doping of bismuth ferrite to tune electromagnetic properties and increase microwave absorption by magnetic-dielectric synergy. J. Mater. Chem. C.

[14] Du Y (2010). Enhancement of ferromagnetic and dielectric properties in lanthanum doped BiFeO_3_ by hydrothermal synthesis. J. Alloy. Comp..

[15] Bao P, Jackson TJ, Wang X, Lancaster MJ (2008). Barium strontium titanate thin film varactors for room-temperature microwave device applications. J. Phys D: Appl. Phys.

[16] Li L (2011). Very large capacitance enhancement in a two-dimensional electron system. Science.

[17] Wang L (2013). Negative quantum capacitance induced by midgap states in single-layer graphene. Sci. Rep..

[18] Luryi S (1988). Quantum capacitance devices. Appl. Phys. Lett..

[19] Riley JM (2015). Negative electronic compressibility and tunable spin splitting in WSe_2_. Nat. Nanotechnol.

[20] Ilani S, Donev LAK, Kindermann M, McEuen PL (2006). Measurement of the quantum capacitance of interacting electrons in carbon nanotubes. Nat. Phys.

[21] He J (2015). Spectroscopic evidence for negative electronic compressibility in a quasi-three-dimensional spin-orbit correlated metal. Nat. Mater..

[22] Santander-Syro AF (2011). Two-dimensional electron gas with universal subbands at the surface of SrTiO_3_. Nature (London).

[23] King PDC (2012). Subband structure of a two-dimensional electron gas formed at the polar surface of the strong spin-orbit perovskite KTaO_3_. Phys. Rev. Lett..

[24] Meevasana W (2011). Creation and control of a two-dimensional electron liquid at the bare SrTiO_3_ surface. Nat. Mater..

[25] Meevasana W (2010). Strong energy-momentum dispersion of phonon-dressed carriers in the lightly doped band insulator SrTiO_3_. New J. Phys..

[26] Masingboon C (2013). Anomalous change in dielectric constant of CaCu_3_ Ti_4_ O_12_ under violet-to-ultraviolet irradiation. Appl. Phys. Lett..

[27] Cheng ZX (2008). Structure, ferroelectric properties, and magnetic properties of the La-doped bismuth ferrite. J. Appl. Phys..

[28] Sen K, Singh K, Gautam A, Singh M (2012). Dispersion studies of La substitution on dielectric and ferroelectric properties of multiferroic BiFeO_3_ ceramic. Ceram. Int..

[29] King PDC (2009). Band gap, electronic structure, and surface electron accumulation of cubic and rhombohedral In_2_ O_3_. Phys. Rev. B.

[30] Zhang L (2005). Electrode and grain-boundary effects on the conductivity of CaCu_3_ Ti_4_ O_12_. Appl. Phys. Lett..

[31] Suwanwong S (2015). The dynamics of ultraviolet-induced oxygen vacancy at the surface of insulating SrTiO_3_ (001). Appl. Surf. Sci..

[32] Mohamad NE, Okimura K, Sakai J (2009). Effect of light irradiation on electric-field-induced resistance switching phenomenon in planar VO_2_ /c-Al_2_ O_3_ structure. Int. J. Nanosci.

[33] Von Hippel, A. R. Dielectrics and Waves. *The MIT Press* (1954).

[34] Aiura Y (2002). Photoemission study of the metallic state of lightly electron-doped SrTiO_3_. Surf. Sci.

[35] Shirley DA (1972). High-resolution X-ray photoemission spectrum of the valence bands of gold. Phys. Rev. B.

[36] Grosvenor AP, Kobe BA, Biesinger MC, McIntyre NS (2004). Investigation of multiplet splitting of Fe 2p XPS spectra and bonding in iron compounds. Surf. Interface anal..

[37] Khan MM (2015). Visible light-induced enhanced photoelectrochemical and photocatalytic studies of gold decorated SnO_2_ nanostructures. New J. Chem..

[38] Wu S, Li S (2014). Light-induced giant capacitance enhancement in LaAlO_3_ /SrTiO_3_ heterostructures. Nanosci. Nanotechnol. Lett.

[39] Zhu M (2016). Capacitance enhancement in a semiconductor nanostructure-based supercapacitor by solar light and a self-powered supercapacitor-photodetector system. Adv. Funct. Mater..

[40] Lei Y (2014). Visible-light-enhanced gating effect at the LaAlO_3_ /SrTiO_3_ interface. Nat. Commun..

[41] Dröscher S., Roulleau P., Molitor F., Studerus P., Stampfer C., Ensslin K., Ihn T. (2010). Quantum capacitance and density of states of graphene. Applied Physics Letters.

[42] Kopp T, Mannhart J (2009). Calculation of the capacitances of conductors: Perspectives for the optimization of electronic devices. J. Appl. Phys..

[43] Skinner B, Shklovskii BI (2010). Anomalously large capacitance of a plane capacitor with a two-dimensional electron gas. Phys. Rev. B.

[44] Larentis S (2014). Band offset and negative compressibility in graphene-MoS_2_ heterostructures. Nano Lett..

[45] Tinkl V, Breitschaft M, Richter C, Mannhart J (2012). Large negative electronic compressibility of LaAlO_3_ -SrTiO_3_ interfaces with ultrathin LaAlO_3_ layers. Phys. Rev. B.

