# Supplementary material for: Interplay of negative electronic compressibility and capacitance enhancement in lightly-doped metal oxide Bi0.95La0.05FeO3 by quantum capacitance model
Source: Sci Rep. 2020 Mar 20;10:5153. doi: 10.1038/s41598-020-61859-6 (PMC7083945; doi:10.1038/s41598-020-61859-6)
Supplement: Supplementary file 1 — Supplementary Information. [file 41598_2020_61859_MOESM1_ESM.pdf]

# Supplementary Information for
# Interplay of negative electronic compressibility and capacitance enhancement in lightly-doped metal oxide $Bi_{0.95}La_{0.05}FeO_3$ by quantum capacitance model

S. Nathabumroong,[1] T. Eknapakul,[1] P. Jaiban,[1, 2] B. Yotburut,[1] S. Siriroj,[1] T. Saisopa,[1] S.-K. Mo,[3] R. Supruangnet,[4] H. Nakajima,[4] R. Yimnirun,[1, 5] S. Maensiri,[1] and W. Meevasana[1, 6, *]

[1]*School of Physics, Suranaree University of Technology, Nakhon Ratchasima, 30000, Thailand*
[2]*Faculty of science, Energy and Environment,*
*King Mongkut's University of Technology North Bangkok,*
*Rayong Campus, Rayong 21120, Thailand*
[3]*Advanced Light Source, Lawrence Berkeley National Laboratory, Berkeley, CA 94720, USA*
[4]*Synchrotron Light Research Institute, Nakhon Ratchasima, 30000 Thailand*
[5]*School of Energy Science and Engineering,*
*Vidyasirimedhi Institute of Science and Technology, Rayong 21210, Thailand*
[6]*NANOTEC-SUT Center of Excellence on Advanced Functional Nanomaterials,*
*Suranaree University of Technology, Nakhon Ratchasima 30000, Thailand*
(Dated: February 1, 2020)

Keywords: La-doped $BiFeO_3$, negative electronic compressibility, quantum capacitance

## I. SAMPLE PREPARATIONS AND CHARACTERIZATIONS

$Bi_{0.95}La_{0.05}FeO_3$ (BLFO) and $BiFeO_3$ (BFO) polycrystals were prepared by a simple co-precipitation method. $[Bi(NO_3)_2{\cdot}5H_2O$, $Fe(NO_3)_2{\cdot}9H_2O$, and $La(NO_3)_3{\cdot}4H_2O$ were dissolved in deionized water under continued stirring. 16 ml of 65% nitric acid ($HNO_3$) was added. Polyethylene glycol (PEG MW=20,000) was then added to promote polymerization at 60 $^oC$. The solution was precipitated using ammonia solution, with pH adjusted to 9.3. The precipitated solution was washed several times by DI water to obtain a pH of 7 and then dried in an oven at 70 $^oC$. BLFO powders were calcined at 600 $^oC$ in air for 3 hrs and then pressed into pellets with 1 cm diameter. BLFO pellets were sintered at 800 $^oC$ in air for 3 hrs to obtain the BFO and BLFO sample; more details are described in [1].

The atomic structure of pure $BiFeO_3$ is rhombohedral with R3c space group (Fig. S1(a)) while substituting $La^{3+}$ ions into $Bi^{3+}$ sites leads to the distortion of their structure to orthorhombic phase [2, 3]. The enhancement of dielectric properties in BLFO can be explained by their electronic structure modification (i.e. doping level, band alignment) and crystal structure modification (i. e. off-centric symmetry preferable, suppression of impurity phases [4]). Our BLFO samples were characterized by scanning electron microscopy (SEM) and X-ray diffraction (XRD) to obtain their morphology and crystal structure. XRD patterns of BLFO and BFO are shown in Fig. S1(b). A small amount of secondary insulating phases such as $Bi_2O_3$ and $Bi_2Fe_4O_9$ is observed in both pure and doped samples. Controlling capacitance of such phases is more difficult compared to the BFO phase hence the changes of capacitance observed in our work should not arise from the impurities. The SEM images enabled grain size determination in our samples which is approximately $5.74 \pm 0.29$ $\mu$m for BFO (Fig. S1(c)). The BLFO grain size is found to be slightly smaller than BFO with more impurities (Fig. S1(d)). The BFO with small doping (i.e. BLFO) exhibits higher capacitance than the pure BFO.

The BLFO samples used for dielectric measurement were polished by sandpaper and diamond paste with acrylic felt to obtain flat surfaces. The polished samples were cleaned in acetone by ultrasonic cleaner. The sample thickness and diameter are approximately 0.8 mm and 4.1 mm, respectively. The dielectric constant of our $Bi_{0.95}La_{0.05}FeO_3$ measured by standard setup (parallel electrode using standard impedance analyzer (Agilent: model 4294A)) is approximately $5{\times}10^4$ at a measured frequency of 1 kHz at room temperature [1]. The dielectric constant of our light irradiation setup is found to be lower than the standard value because of poor electrical contact and air gap.

*Corresponding e-mail: worawat@g.sut.ac.th

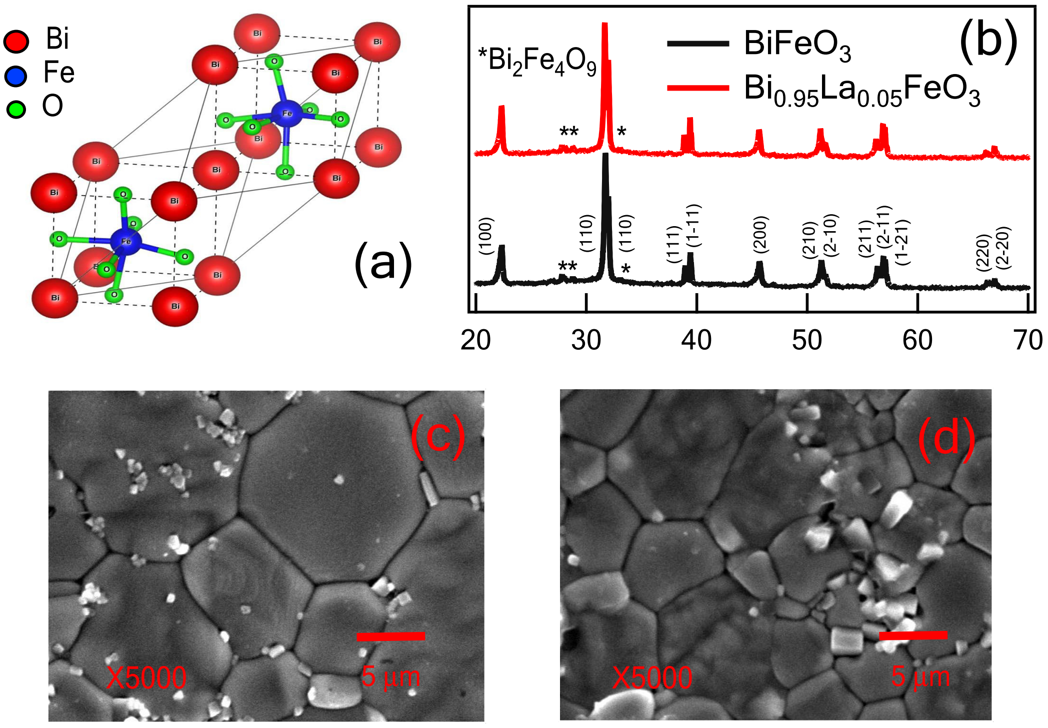


FIG. S1: a) Atomic structure of $BiFeO_3$. b) XRD patterns and c)-d) SEM images of BFO and BLFO samples, respectively.

## II. DETERMINING ULTRAVIOLET PHOTOEMISSION SPECTRA (UPS) AND THE EXTRACTION OF $O_{2p}$ VALENCE STATE UNDER UV IRRADIATION

In this study, the evolution of the $O_{2p}$ valence state of the BLFO sample under UV irradiation was measured by ultraviolet photoemission spectroscopy (UPS). As shown in Fig. S2(a), it is clearly seen that the $O_{2p}$ valence state shifts to lower binding energy by increasing irradiation time. To confirm this effect, the shift of $O_{2p}$ peak position would be the main evidence representing the modification of BLFO electronic structure under light irradiation. To determine the $O_{2p}$ peak positions, UPS spectra were firstly subtracted by Shirley background to remove an extrinsic loss structure [5–7]. After that, the processed spectra were fitted by Gaussian equation and the peak positions could be obtained. The fitting equation can be expressed by the following:

$$f(x) \;=\; SB \;+\; A_0 exp[-(\frac{x - x_0}{w})^2] \tag{1}$$

where, SB is Shirley background, $A_0$ is amplitude, $x_0$ is $O_{2p}$ peak position and $w$ is full width at half maximum (FWHM). An example of UPS fitting is shown in Fig. S2(b) where black, orange, and green curves are the measured UPS spectra, Gaussian fitted curve, and Shirley background (SB), respectively.

## III. CALCULATION OF LIGHT DOSE

In this work, violet laser and UV synchrotron radiation have been used as light sources. Light dose (D) is a crucial parameter to study the irradiation effect which can be calculated using the same approach for both sources as follows:

$$D \;=\; It \tag{2}$$

where I is intensity ($W{\cdot}cm^{-2}$) and t is exposure time (s)

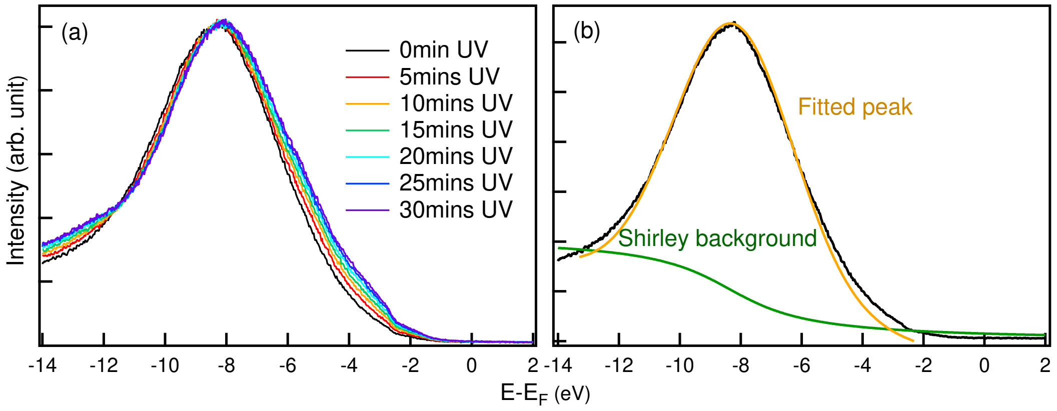


FIG. S2: a) Evolution of the $O_{2p}$ valence state of the BLFO pellet as a function of UV irradiation. b) The example of Gaussian fitting at $O_{2p}$ valence state.

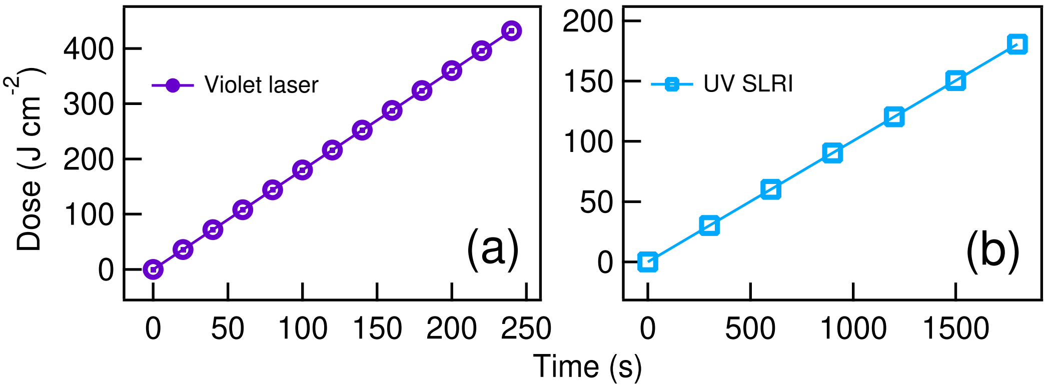


FIG. S3: The calculated light dose acquired by a) violet laser and b) UV synchrotron irradiation.

## A. Violet laser

The violet laser intensity ($I_v$) was measured directly by conventional photometer (Thorlab: model PM100D). In this work, $I_v$ was fixed to be 0.18 W·cm$^{-2}$ throughout experiments and a light dose as a function of time can be calculated as shown in Fig. S3(a).

## B. UV synchrotron radiation

The ultraviolet light radiation was measured at Synchrotron Light Research Institute (SLRI), Thailand. From standard synchrotron radiation, synchrotron light intensity ($I_s$) can be calculated from the following equation:

$$I_s \;=\; h\nu \cdot \frac{1}{d_b} \cdot P_\phi \tag{3}$$

where h$\nu$ is incident photon energy which was set to be 60 eV, $d_b$ (beam size) is estimated to be around 0.3 mm x 0.1 mm. The $P_\phi$ stands for number of photons per second (photon flux) which was calculated from photon fluence ($\phi$) by the following method.

$\phi$ is defined by the number of photons (dN) that enter an imaginary space of cross-sectional area (dA) given by:

$$\phi \;=\; \frac{dN}{dA} \tag{4}$$

By definition, photon fluence can be calculated by the multiplication of the number of photons per second ($D_\phi$) impacting to the unit area of the sample and the irradiation time (t).

$$\phi = D_\phi t \tag{5}$$

$D_\phi$ can be calculated by:

$$D_\phi = \frac{P_\phi}{A} \tag{6}$$

where A is the exposed areas of the sample. In this measurement, $P_\phi$ can be obtained by the photoelectrons per unit time ($I_p$) per the quantum efficiency ($Q_p$) of the photodiodes at a given photon energy:

$$P_\phi = \frac{I_p}{eQ_p} \tag{7}$$

In our measurement, $P_\phi$ can be obtained by measuring the gold mesh photocurrent ($I_g$) which is located before UPS chamber of BL3.2a at SLRI. The standard gold mesh was used to monitor the real-time photocurrent during UPS. Based on our calibration, photon flux can be estimated by:

$$P_\phi = \frac{I_g}{0.65eQ_g} \tag{8}$$

where $Q_g = 0.04$ is the quantum efficiency of the gold mesh, $I_g = 13$ nA and $P_\phi$ was calculated to be $3.13 \times 10^{12}$ photons/s. By substituting all parameters into eq. S3, the synchrotron light dose as a function of irradiation time was obtained as shown in Fig. S3(b)

## IV. DETERMINING $N_{2D}$ BY UPS SPECTRA

Regarding light irradiation, two-dimensional electrons gases (2DEGs) were expected to form on BLFO surfaces resulting in the creation of surface two-dimensional electron density ($n_{2D}$) [8]. The $n_{2D}$ value can be estimated by the following approach.

We begin with the calculated quantum capacitance ($C_q$) which can be expressed as [9]:

$$C_q = Ae^2\frac{dn}{d\mu} \tag{9}$$

$C_q$ can be calculated from an increasing of overall capacitance by an inverse formula of two capacitors connecting in series (i.e. $\frac{1}{C_{tot}} = \frac{1}{C_{geo}} + \frac{1}{C_q}$).

Eq. S9 can be modified to:

$$dn = \frac{C_q \cdot edV}{Ae^2} \quad ;where \;\; d\mu = edV \tag{10}$$

Then, we multiply light dose (dD) term to both sides of the above equation, as a result, dn can be expressed as a function of dosing. We assume that dn is accumulated on very thin surface, hence, in this case dn $\approx$ $dn_{2D}$.

$$\frac{Cq \cdot \frac{dV}{dD} \cdot dD}{Ae} = dn_{2D}(D) \tag{11}$$

By integrating the above equation, $n_{2D}(D)$ can be calculated by:

$$n_{2D}(D) \approx \int dn_{dD} = \int \frac{C_q \frac{dV}{dD}}{Ae} \cdot dD \tag{12}$$

where $n_{2D}$(D) represents the electron density as a function of light dose. The calculated $n_{2D}$ (D) is shown in Fig. S4 (similar to Fig. 3(b) in the manuscript)

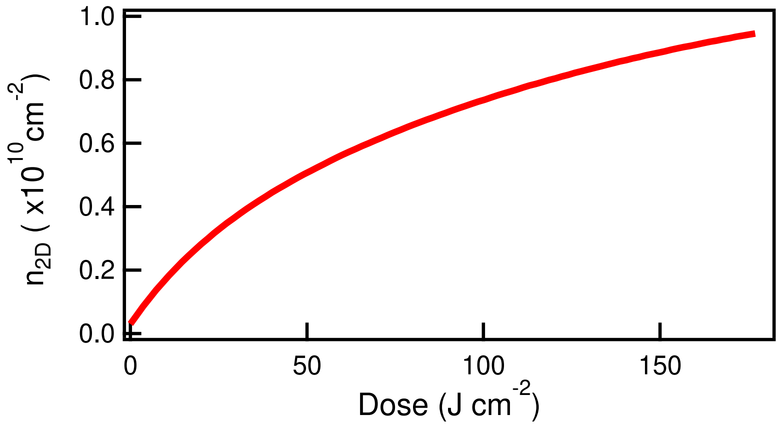


FIG. S4: Two-dimensional electron density ($n_{2D}$) as a function of light dose.

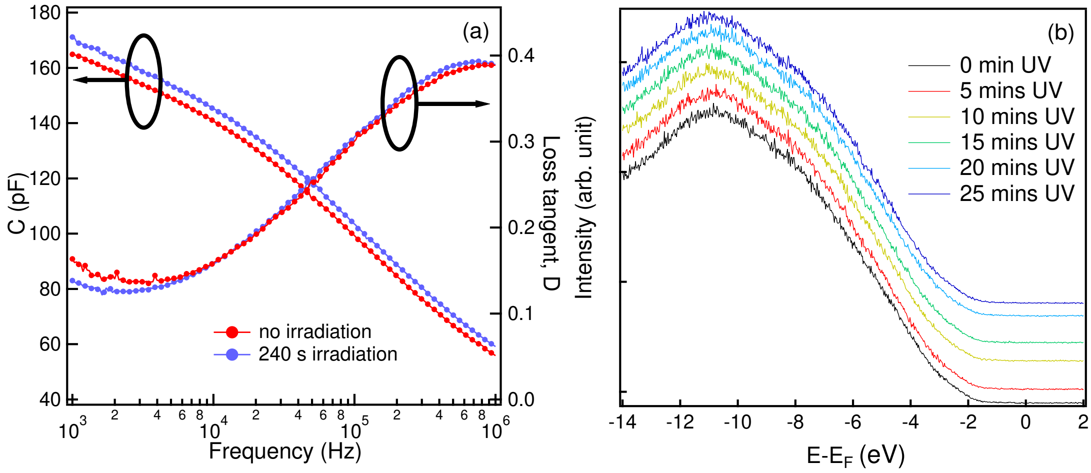


FIG. S5: Frequency dependence of capacitance and loss tangent with and without violet laser irradiation. b) UPS spectra of BFO measured before and after UV irradiation.

## V. CAPACITANCE MEASUREMENT AND UPS SPECTRA OF BIFEO$_3$

We have performed the capacitance measurement and ultraviolet photoemission spectroscopy of BFO as shown in Fig. S5. Frequency dependent measurements of capacitance and loss tangent before and after irradiation are shown in Fig. S5(a). We found that the dielectric behavior of BFO is different from BLFO [10, 11]. After 240 s irradiation, the capacitance enhancement can be found at all frequency, while, loss tangent increases at low frequency and then slightly decreases at high frequency. The UPS spectra of BFO under UV irradiation is shown in Fig. S5(b). After UV irradiation, a shift of $O_{2p}$ valence state to lower binding energy (extracted value up to 40 $\pm$15 meV) was observed but was around 5 times smaller than BLFO. The maximum capacitance enhancement in BLFO is around 4 times higher than BFO.

## VI. DIELECTRIC CONSTANT AND THE INCREASE OF IMAGINARY PART OF THE PERMITTIVITY OF BIFEO$_3$ UNDER IRRADIATION

Dielectric constant of BLFO can be calculated from geometric capacitance ($C = \epsilon\frac{A}{d}$), where A is sample surface area (A = 12.56 mm$^2$) and d is sample thickness (d = 0.8 mm). The frequency dependence of BLFO dielectric constant before and after irradiation are shown in Fig. S6a. Because of poor electrical contact and air gap, the dielectric constant of our light irradiation setup is found to be lower than the standard value which is approximately 5$\times$10$^4$ at a measured frequency of 1

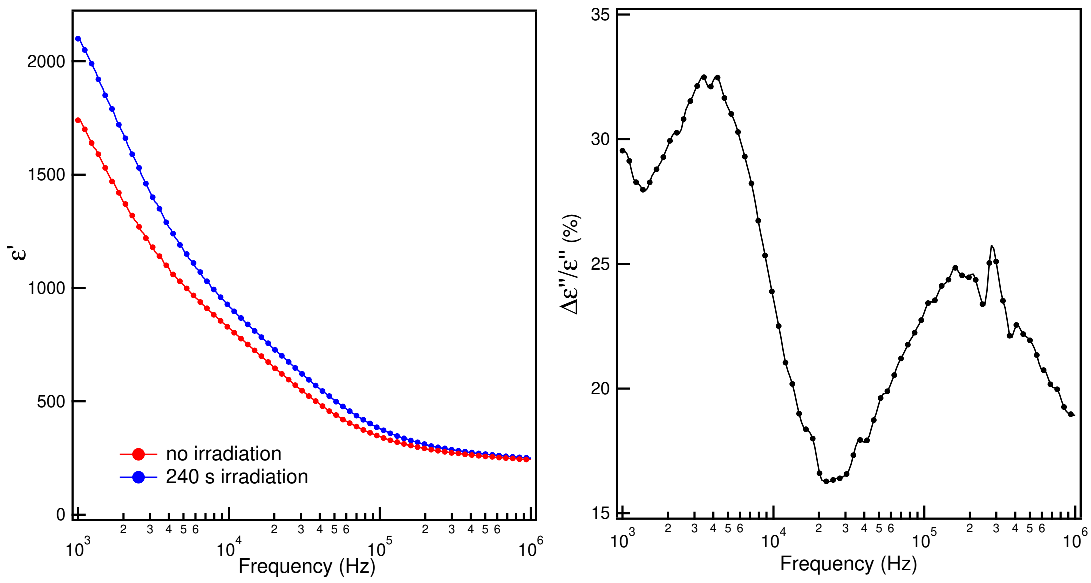


FIG. S6: a) Frequency dependence of dielectric constant and b) the increase of imaginary part of the permittivity after 240 s irradiation

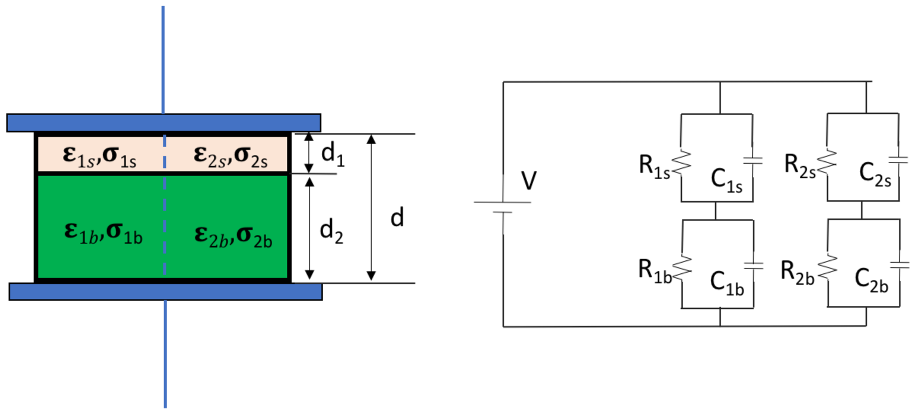


FIG. S7: Schematic model of two-layer heterogeneous system of bulk and surface and its equivalent circuit. Note that $\varepsilon_{1b} = \varepsilon_{2b}$ and $\sigma_{1b} = \sigma_{2b}$ (bulk values are the same); $\varepsilon_{1s}$ and $\varepsilon_{2s}$ represent dielectrics of grain and grain boundary at the surface, respectively.

kHz at room temperature [1]. We note that low dielectric constant measured by our setup does not affect to the quantity of quantum capacitance. The increase of imaginary part of the permittivity ($\epsilon'' = \epsilon' \times D$) have been shown in Fig. S6b which indicates different shape compared to the capacitance enhancement.

## VII. MAXWELL-WAGNER AND QUANTUM CAPACITANCE MODEL

We utilized the two-independent Maxwell-Wagner (MW) model of two-layer heterogeneous systems (i.e. grain and grain boundary) to address the effect of light irradiation. The schematic model and its equivalent circuit are shown in Fig. S7. Following the analysis presented in [12], the two independent MW equation could then be expressed as [13]

$$\varepsilon' = \frac{\tau_{1b} + \tau_{1s} - \tau_{1t} + \omega^2 \tau_{1b}\tau_{1s}\tau_{1t}}{C_0\,(R_{1b} + R_{1s})\,(1 + \omega^2 \tau_{1t}^2)} + \frac{\tau_{2b} + \tau_{2s} - \tau_{2t} + \omega^2 \tau_{2b}\tau_{2s}\tau_{2t}}{C_0\,(R_{2b} + R_{2s})\,(1 + \omega^2 \tau_{2t}^2)} \tag{13}$$

Where $\varepsilon'$= total dielectric constant, $\omega = 2\pi f, R_{1s} = \rho_{1s}\frac{d_{1s}}{A}, C_{1s} = \varepsilon_{1s}\frac{A}{d_{1s}}, \tau_{1s} = \rho_{1s}\varepsilon_{1s}$. $\rho_{1s}$ and

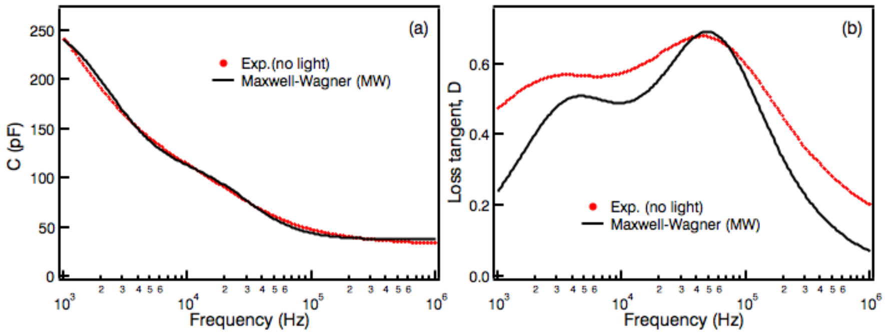


FIG. S8: a) Capacitance and b) loss tangent taken from experiment (red-dotted line) and Maxwell-Wagner model (black curve) as a function of frequency before shining the light.

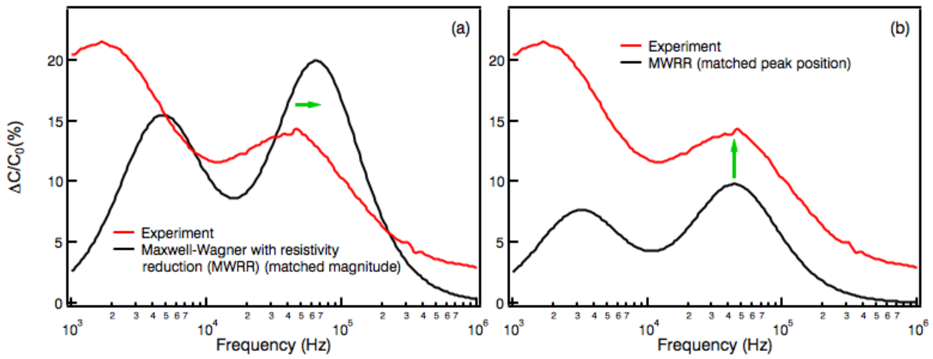


FIG. S9: Calculated capacitance enhancement using Maxwell-Wagner with resistivity reduction a) matched magnitude b) matched peak position.

$\rho_{1b}$ are surface and bulk resistivity. $\varepsilon_{1s}$ and $\varepsilon_{1b}$ are surface and bulk permittivity. $\tau_{1t} = \frac{\varepsilon_{1b} d_{1s} + \varepsilon_{1s} d_{1b}}{\frac{d_{1s}}{\rho_{1b}} + \frac{d_{1b}}{\rho_{1s}}}$ and d = $d_s + d_b$. *Subscript 1 and 2 refer to grain and grain boundary. **Subscript s and b refer to surface and bulk. It is known that light can penetrate into the sample within a few microns [14] and electrons can only be confined in the range between 2-4 nm near the surface [15]. In our model, we assume that light can penetrate into the BLFO surface with the upper limit of 8 $\mu$m ($d_s$/d=0.01) which therefore lower the resistivity of the surface.

Starting from the measurements before shining the light, the capacitance and loss tangent as a function of frequency can be fit well as shown in Fig. S8. However, after shining the light, we find that it is not possible to fit the measured data without invoking the negative electronic compressibility model.

As shown in Fig. S9, while the reduction of surface resistivity (i.e. due to suggested photogeneration of charge carrier) could help enhancing the capacitance (see black-color line); the line shape does not match. In the case of increasing the magnitude, the two peaks of the characteristic frequencies will shift (Fig. S9(a)) but if we match the two characteristic peaks, the magnitude will be too low (Fig. S9(b)). However, after incorporating both the surface resistivity change and the NEC effect, we get the nicely-fit data as depicted in Fig. S10 (Fig. 1(g) of the manuscript). This additional enhancement also matches in the order of magnitude with the negative capacitance calculated from the negative energy shift measured by the photoemission spectroscopy in Fig. 2(b) of the manuscript, strongly supporting that the NEC effect plays the important role in this enhancement.

[1] Yotburut, B., Yamwong, T., Thongbai, P. & Maensiri, S. Synthesis and characterization of coprecipitation-prepared La-doped $BiFeO_3$ nanopowders and their bulk dielectric properties. *Jpn. J.*

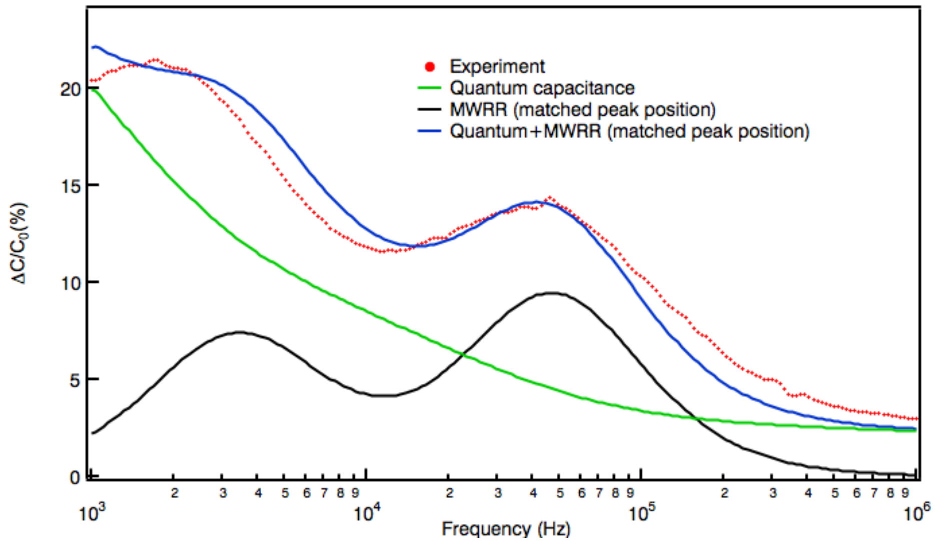


FIG. S10: The change of capacitance after 240 s irradiation (red-dotted curve). Green and black curves represent the calculated capacitance enhancement using quantum capacitance and Maxwell-Wagner model, respectively. The summation of these model is represented by blue curve which is in agreement to the observed capacitance enhancement by light irradiation.

*Appl. Phys.* **53**, 06JG13 (2014).
[2] Du, Y. et al. Enhancement of ferromagnetic and dielectric properties in lanthanum doped $BiFeO_3$ by hydrothermal synthesis *J. Alloy. Comp.* **490**, 637 (2010).
[3] Zheng, X. et al. The magnetic properties of La doped and codoped $BiFeO_3$. *J. Alloy. Comp.* **499**, 108 (2010).
[4] Chaudhuri, A & Mandal, K. Enhancement of ferromagnetic and dielectric properties of lanthanum doped bismuth ferrite nanostructures. *Mater. Res. Bull.* **47**, 1057 (2012).
[5] Shirley, D. A. High-resolution X-ray photoemission spectrum of the valence bands of gold. *Phys. Rev. B* **5**, 4709 (1972).
[6] Grosvenor, A. P., Kobe, B. A., Biesinger, M. C. & McIntyre, N. S. Investigation of multiplet splitting of Fe 2p XPS spectra and bonding in iron compounds. *Surf. Interface anal.* **36**, 1564-1574 (2004).
[7] Norgren, B. S., Somers, M. A. J. & De Wit, J. H. W. Application of tougaard background subtraction to XPS spectra of passivated Fe-17 Cr. *Surf. Interface Anal.* **21**, 378 (1994).
[8] Lei, Y. et al. Visible-light-enhanced gating effect at the $LaAlO_3/SrTiO_3$ interface. *Nat. Commun.* **5**, 5554 (2014).
[9] Li, L. et al. Very large capacitance enhancement in a two-dimensional electron system. *Science* **332**, 825-828 (2011).
[10] Palaimiene, E. Macutkevic, J., Karpinsky, D. V., Kholkin, A. L. & Banys, J. Dielectric investigations of polycrystalline samarium bismuth ferrite ceramic. *Appl. Phys. Lett.* **106**, 012906 (2015)
[11] Lia, W. & Schwartz, R. W. Dielectric response of Sr doped $CaCu_3Ti_4O_{12}$ ceramics. *Appl. Phys. Lett.* **90**, 112901 (2007).
[12] Von Hippel, A. R. Dielectrics and Waves, *The MIT Press*, (1954).
[13] Prodromakis, T. & Papavassiliou C. Engineering the Maxwell-Wagner polarization effect. *Appl. Surf. Sci.* **255**, 69896994 (2009).
[14] Land, C. E. & Peercy, P. S. A review of the effects of ion implantation on the photoferroelectrics properties of PLZT ceramics. *Ferroelectrics* **45**, 25 (1982).
[15] Meevasana, W. et al. Creation and control of a two-dimensional electron liquid at the bare $SrTiO_3$ surface. *Nat. Mater.* **10**, 114 (2011).
